# Precision Grinding Technology of Silicon Carbide (SiC) Ceramics by Longitudinal Torsional Ultrasonic Vibrations

**DOI:** 10.3390/ma16165572

**Published:** 2023-08-10

**Authors:** Zejiu Ye, Xu Wen, Weiqiang Wan, Fuchu Liu, Wei Bai, Chao Xu, Hui Chen, Pan Gong, Guangchao Han

**Affiliations:** 1 School of Mechanical Engineering and Electronic Information, China University of Geosciences, Wuhan 430074, China; 2 Shenzhen Research Institute, China University of Geosciences, Shenzhen 518063, China; 3 Shenzhen Tsingding Technology Co., Ltd., Shenzhen 518108, China; 4 Division of Advanced Manufacturing, Shenzhen International Graduate School, Tsinghua University, Shenzhen 518055, China; 5 State Key Laboratory of Solidification Processing, Northwestern Polytechnical University, Xi’an 710072, China; hchen1984@nwpu.edu.cn; 6 State Key Laboratory of Materials Processing and Die & Mould Technology, School of Materials Science and Engineering, Huazhong University of Science and Technology, Wuhan 430074, China; pangong@mail.hust.edu.cn

**Keywords:** longitudinal torsional ultrasonic vibrations, silicon carbide ceramics, grinding removal mechanism, cutting force prediction model, process parameter optimization

## Abstract

Silicon carbide (SiC) ceramic material has become the most promising third-generation semiconductor material for its excellent mechanical properties at room temperature and high temperature. However, SiC ceramic machining has serious tool wear, low machining efficiency, poor machining quality and other disadvantages due to its high hardness and high wear resistance, which limits the promotion and application of such materials. In this paper, comparison experiments of longitudinal torsional ultrasonic vibration grinding (LTUVG) and common grinding (CG) of SiC ceramics were conducted, and the longitudinal torsional ultrasonic vibration grinding SiC ceramics cutting force model was developed. In addition, the effects of ultrasonic machining parameters on cutting forces, machining quality and subsurface cracking were investigated, and the main factors and optimal parameters affecting the cutting force improvement rate were obtained by orthogonal tests. The results showed that the maximum improvement of cutting force, surface roughness and subsurface crack fracture depth by longitudinal torsional ultrasonic vibrations were 82.59%, 22.78% and 30.75%, respectively. A longitudinal torsional ultrasonic vibrations cutting force prediction model containing the parameters of tool, material properties and ultrasound was established by the removal characteristics of SiC ceramic material, ultrasonic grinding principle and brittle fracture theory. And the predicted results were in good agreement with the experimental results, and the maximum error was less than 15%. The optimum process parameters for cutting force reduction were a spindle speed of 22,000 rpm, a feed rate of 600 mm/min and a depth of cut of 0.011 mm.

## 1. Introduction

**Figure 1:**
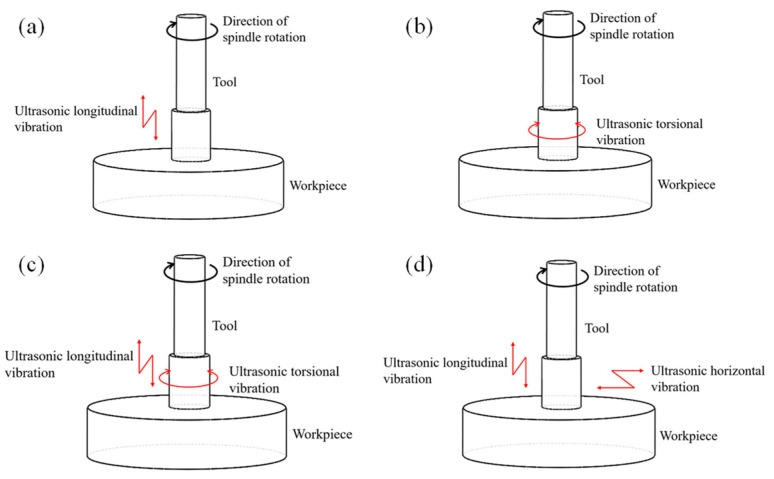
Different ultrasonic vibration modes of the tool in ultrasonic-assisted milling: (**a**) ultrasonic longitudinal vibration, (**b**) ultrasonic torsional vibration, (**c**) ultrasonic longitudinal torsional vibration and (**d**) ultrasonic elliptical vibration.

In recent years, the ultrasonic grinding technology of hard and brittle materials has been developed rapidly [1,2,3]. Ultrasonic grinding is the application of ultrasonic vibration on the grinding head or workpiece, so that the grinding head and the workpiece achieve ultrasonic frequency intermittent processing, so as to effectively reduce the friction between the grinding head and the machining surface, reduce the heat accumulation during the processing process, reduce the chip sticking on the grinding particle caused by blockage, resulting in wear of the grinding head, and improve the surface quality of the machined. Compared with traditional grinding, its processing efficiency and processing quality have been greatly improved. At present, the commonly used ultrasonic vibration modes mainly include axial vibration, torsional vibration, longitudinal torsional ultrasonic vibrations and elliptical vibration, as shown in Figure 1.

**Figure 2:**
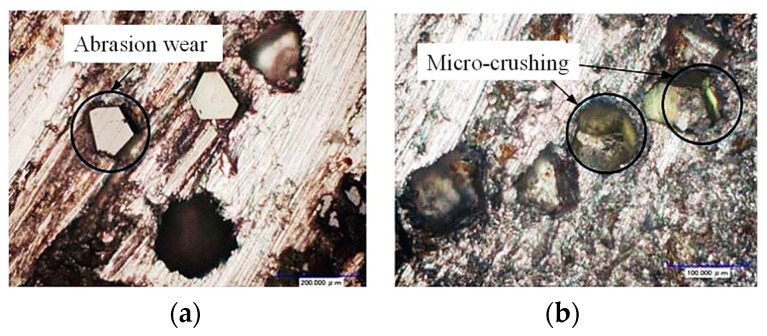
End face morphology of the diamond tool [19]: (**a**) common processing and (**b**) rotary ultrasonic processing.

Ultrasonic machining techniques have been gradually applied to the machining of SiC ceramic materials. As one of the most representative hard and brittle materials, many scholars have studied the ultrasonic grinding properties of SiC ceramics. These studies focus on the grinding force, material removal mechanism, surface and subsurface quality, and tool wear of SiC ceramic ultrasonic grinding. Zhang et al. conducted a comparison experiment between elliptical ultrasonic-vibration-assisted grinding (EUVAG) and conventional grinding (CG) on single-crystal SiC ceramics. The experimental results showed that EUVAG had a significant reduction in grinding force and specific energy and improved material removal rate compared with CG [4,5,6]. Wang et al. explored the typical damage forms of C/SiC composites during end and side grinding. The degree of surface and subsurface damage of C/SiC composites during grinding and ultrasonic-vibration-assisted grinding was compared. The results showed that the damage forms were essentially the same for plain grinding and ultrasonic grinding. Compared with normal grinding, ultrasonic-assisted grinding can reduce surface damage to a certain extent and significantly reduce subsurface damage [7]. Wang et al. conducted a comparative experimental study of ultrasonic-vibration-assisted grinding and ordinary grinding on C/SiC composites; the variation laws of grinding force and grinding force ratio under different processing methods and process parameters were analyzed. The results showed that ultrasonic vibration can soften the C/SiC material to a certain extent through the effect of high-frequency impact, sharpen the cutting edge, greatly reduce the grinding force value and improve the machinability of the material [8]. Dai et al. studied the structure of a single abrasive particle and the effect of wear on the removal mechanism of SIC ceramics. It has been shown that the main form of wear on a single abrasive particle is back tool surface wear, and that increasing the grinding radius of the abrasive particle results in better machined surface quality [9]. Cao et al. studied the simulation of single abrasive particle scratching of SiC ceramics and conducted the ultrasonic vibration scratching test of a single abrasive particle. The results show that there are two forms of material removal with varying cut depth during ultrasonic vibration scratching, namely intermittent cutting mode and continuous cutting mode. The common scratching process is a continuous cutting pattern. Ultrasonic vibrational scraping of SiC brittle–plastic transition cuts deeper than ordinary scraping [10]. Ding et al. designed a wheel with fixed structure to conduct a comparative test study on ultrasonic vibration grinding of SiC ceramics and ordinary grinding, and mainly studied the removal mechanism of SiC ceramics in the grinding process. The results show that the removal pattern of SiC ceramic materials changes from plastic removal to brittle fracture removal as the cut depth increases [11]. Zhou et al. carried out ultrasound-assisted scratching tests on BK7 and JGS1 glass with a diamond indenting head, and the results showed that ultrasonic vibration could significantly increase the critical cutting depth of the brittle–plastic transition of the glass material, so ultrasound-assisted grinding is easier to achieve plastic-domain grinding [12]. Liang et al. studied the scratch micro-morphology, cross section depth and width size of single-crystal sapphire material marked by ultrasonic-assisted elliptic vibration of a single diamond abrasive particle, and also believed that ultrasonic vibration could significantly increase the critical thickness of the brittle–plastic transition [13]. Xu et al. conducted an ultrasonic vibration grinding test on SiC ceramics to study the influence of cutting force and workpiece surface quality in ultrasonic vibration grinding. The test results show that the introduction of ultrasound vibrations with the same process parameters is beneficial in reducing the grinding force, resulting in a more uniform surface topography and better surface quality [14]. Pradhan et al. set up a complex frequency ultrasonic machining test system and conducted a complex frequency ultrasonic drilling test for a SiC ceramic plate. Experimental studies have shown that changing the free mass block and the axial electrostatic pressure can effectively increase the machining efficiency by about eight times compared to conventional ultrasonic machining [15]. Cao et al. compared the inner-circle ultrasonic-assisted grinding of SiC materials with conventional grinding and concluded that compared with conventional grinding, ultrasonic-assisted grinding has achieved greater improvement in shape accuracy and surface roughness, and the wear marks and grinding cracks on the working surface can be greatly suppressed [16]. Zeng et al. studied the change of tool wear of SiC and alumina ceramics during ultrasonic vibration grinding to make holes. The experimental results show that the first stage of crushing wear can be effectively avoided in the milling process, as there is no need to sharpen before machining. The main wear was wear and abrasive drop wear, and it was found that the end face of the grinding head was more heavily worn than the side face. Wear on the head can be reduced by designing a specific structure for the head, that is, reducing the height of the diamond particles on the side of the head [17,18]. Ding et al. studied the grinding head wear characteristics of ultrasonic grinding and ordinary grinding of SiC ceramics, as shown in Figure 2. It was shown that the main type of head wear during ultrasonic vibration grinding was abrasive crushing, which was similar to grinding wheel sharpening and contributes to grinding power. The main type of wear on the grinding head in common grinding processes is wear [19].

In summary, it can be seen that most of the research on ultrasonic vibration grinding of SiC ceramic materials has focused on rotating ultrasonic vibration grinding and the effect of ultrasonic vibration on grinding force and surface quality. Research on other forms of ultrasonic vibration grinding and its surface creation mechanism still needs to be further enhanced. The difficulties in processing SiC ceramic materials are mainly in the following areas [20,21]:

(1) High requirements for surface integrity of the processed material. With regular milling and wheel milling, there are often fine surface cracks, broken material edges and poor surface roughness.
(2) Low processing efficiency. SiC has a Mohs hardness of 9.25–9.5; with the traditional chemical mechanical polishing (CMP) to remove material to a 1–2 μm depth, it takes tens of hours to complete.
(3) Hard and brittle material process performance is poor. SiC is increasingly in demand as a hard and brittle material in new energy vehicles, optics, smart grid and other applications. These materials have excellent mechanical properties but also imply difficulties in processing, and the current processing process for hard and brittle materials is not yet mature.

## 2. Cutting Force Modeling

The cutting force during the SiC ceramic grinding process can effectively reflect the interaction state between the grinding head and the workpiece, as well as visually reflect the material and tool changes during the machining process. The magnitude of the grinding force directly affects the quality of the machined surface and the state of wear on the abrasive grains of the head. Therefore, the research of cutting force in machining has been the key concern of scholars. The magnitude of the cutting force during the milling process is mainly related to the physical properties of the material to be processed, the mesh and structure of the grinding head, the selection of the process parameters, the cooling conditions used during the machining process and the performance structure of the machining center. The material removal mechanism of SiC ceramic materials in the grinding process is complicated. Currently, some results have been achieved in the study of the cutting force during grinding, but a model for the prediction of the cutting force for longitudinal torsional composite ultrasonic vibration grinding of SiC ceramic materials is lacking. This section theoretically models the cutting force for longitudinal torsional composite ultrasonic vibration grinding of SiC ceramic materials by combining the theory of removal properties of SiC ceramics, the principles of grinding and the fracture mechanics of brittle materials.

### 2.1. Study on Material Removal Mechanism of Ceramic Materials

**Figure 3:**
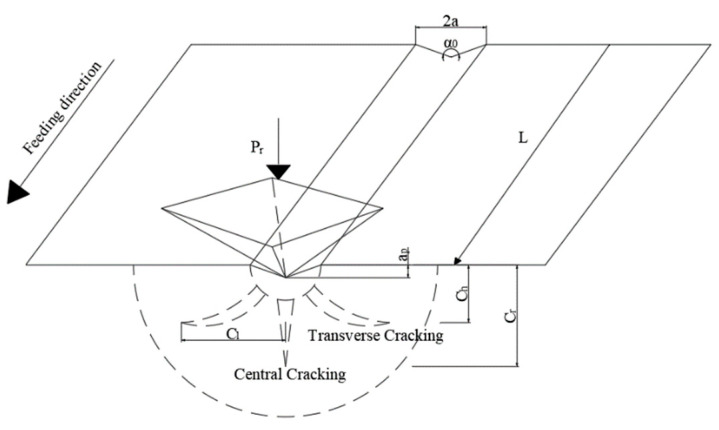
Scratching diagram.

Longitudinal torsional compound ultrasonic vibration grinding of brittle materials produces moderate radial and transverse cracks as diamond particles are pressed against the surface of the workpiece. The sprouting and expansion of these radial and transverse cracks eventually results in chipping of the workpiece, which results in the material being removed in a brittle manner. As shown in Figure 3, the diamond grains move on the surface with velocity V_0_; with respect to the workpiece, and the abrasive grains are subjected mainly to normal and tangential forces during their motion. $F_{n}$ represents the normal force, $F_{t}$ represents the tangential force, $a_{p}$ is the cutting depth of the abrasive grain, $\alpha_{0}$ is the cone angle of the abrasive grain and 2a is the width of the groove caused by the abrasive grain scratching. From the geometrical relations in Figure 3 we can obtain:

(1) $$2a=2a_{p}\times \tan\left(\alpha_{0}/2\right)$$

The contact surface between the abrasive grain and the workpiece during the grinding process is only two sides, so the relationship between the normal force $F_{n}$ and 2a is shown in Equation (2),

(2) $$F_{n}=\frac{1}{2}\delta Ha^{2}$$

where δ is the Vickers indenter geometry factor, δ = 1.8544; H is the Vickers hardness of the ceramic material.

According to Equations (1) and (2), the normal force $F_{n}$ was obtained, as shown in Equation (3).

(3) $$F_{n}=\frac{1}{2}\delta a_{p}^{2}\tan^{2}\left(a_{0}∕2\right)H$$

According to the fracture mechanics theory [22], the transverse crack depth $C_{l}$, transverse crack depth $C_{h}$ and central crack depth $C_{r}$ are calculated as shown in Equations (4)–(6):

(4) $$C_{l}=m{\left(\frac{1}{\tan\left(\alpha_{0}/2\right)}\right)}^{\frac{5}{12}}\cdot {\left(\frac{E^{\frac{3}{4}}}{HK_{IC}{\left(1-v^{2}\right)}^{\frac{1}{2}}}\right)}^{\frac{1}{2}}\cdot F_{n}^{\frac{5}{8}}$$

(5) $$C_{h}=0.43{\left(\sin\alpha_{0}\right)}^{\frac{1}{2}}{\left(\cot\alpha_{0}\right)}^{\frac{1}{3}}{\left(\frac{E}{H}\right)}^{m}{\left(\frac{F_{n}}{H}\right)}^{\frac{1}{2}}$$

(6) $$C_{r}=\alpha_{K}^{\frac{2}{3}}{\left(\cot\alpha_{0}\right)}^{\frac{4}{9}}{\left(\frac{E}{H}\right)}^{{\left(1-m\right)}^{\frac{2}{3}}}{\left(\frac{F_{n}}{K_{IC}}\right)}^{\frac{2}{3}}$$

where v is the Poisson’s ratio of the material; E is the modulus of elasticity of the material; m is a dimensionless constant between 1/3 and 1/2; $K_{IC}$ is the fracture toughness of the material; and $a_{K}$ is the proportionality coefficient, where $a_{K}$ = 0.027 + 0.090 (m^−1/3^).

### 2.2. Analysis of the Cutting Force Model

**Figure 4:**
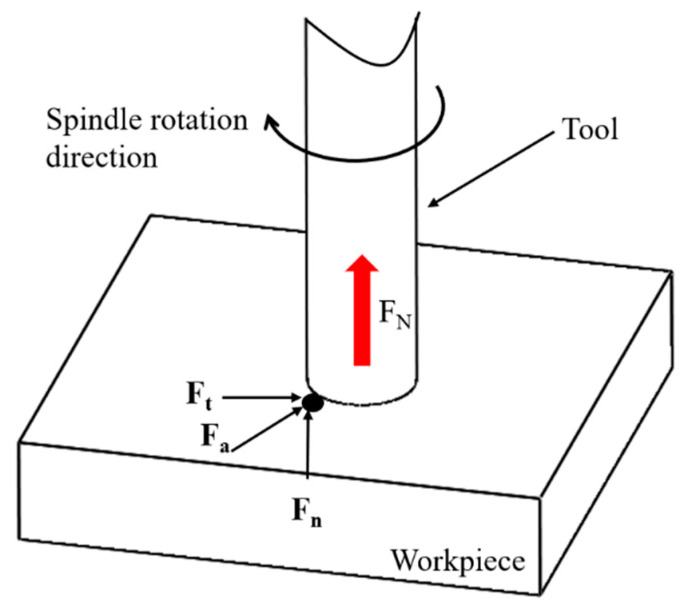
Diagram of the force on diamond grains on the grinding head. ( The arrow of the cutting force in the figure represents the direction of the force).

In the longitudinal torsional compound ultrasonic vibrational grinding process, material removal is primarily accomplished by the vibrational action of diamond abrasive grains on the end face of the grinding head. The force exerted on a diamond grain on a grinding head is shown in Figure 4. The cutting force on a single diamond grain can be divided into three main parts: axial force $F_{n}$, radial force $F_{a}$ and tangential force $F_{t}$. Due to the symmetry of the diamond tools during machining, the radial and tangential forces to which they are subjected are small and negligible. Therefore, the modeling in this section focuses on the cutting force$\;F_{n}$.

The model is based on the following simplifications and assumptions:

(1) The diamond abrasive particles on the surface of the grinding head are evenly distributed; the size is the same, and the exposed height of the abrasive particles is equal; and the diamond abrasive particles are approximately seen as a rigid regular octahedron.
(2) The diamond abrasive particles on the surface of the grinding head are involved in the grinding process.
(3) The SiC ceramic material is removed in accordance with the brittle fracture mode.

### 2.3. Establishment of the Cutting Force Model

In ultrasonic vibration grinding of brittle materials, abrasive grains remove material from the surface of the workpiece through a mixture of diamond grain processing caused by rotation of the diamond tool, ultrasonic impact and ultrasonic-vibration-induced wear and impact. Thus, the volume of material removed by a single diamond grain in a single vibrational cycle can be determined by the transverse crack length and depth, as well as the distance the grain is in contact with the workpiece in a single vibrational cycle. In order to simplify the calculation of the material removal volume, the actual material removal volume due to a diamond particle is defined in this paper as proportional to the theoretical volume of the fracture removal zone during one vibration cycle. From this, the material removal volume V_0_ of a diamond particle in one vibrational period is given in Equation (7), where K is the scaling parameter, which can be obtained by designing the experiment for a given workpiece material, and L is the effective cut length of the abrasive grain.

(7) $$V=2K\times C_{l}\times C_{h}\times L$$

**Figure 5:**
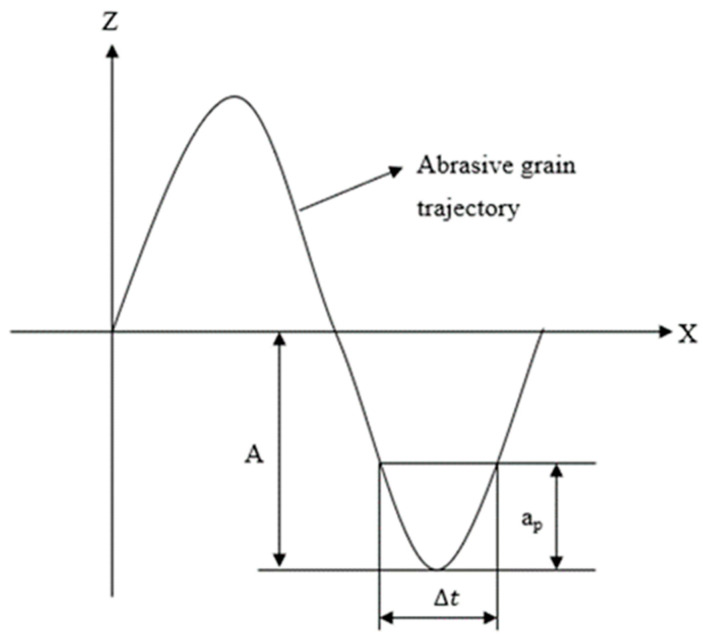
Simplified movement of diamond grains in longitudinal torsion compound ultrasonic processing.

Since the position of the abrasive grains on the end face of the diamond tool is different, to simplify the calculation, L can be calculated by Equation (8), where r is the radius of the diamond grinding head; n is the spindle speed; Δt is the actual contact time between a single diamond abrasive grain and the material during one vibration cycle of ultrasonic processing, As shown in Figure 5, Δt = $a_{p}$/2Af, where $a_{p}$ is the depth of the diamond abrasive grain pressed into the material; and A is the amplitude of the ultrasound amplitude.

(8) $$L=\frac{2\pi rn}{60}\Delta t$$

The maximum load on the grinding head $F_{c}$ can be expressed by Equation (9), where m is the number of diamond grains involved in grinding. In the above cut force modeling, assumptions were made for the abrasive grain of the diamond head, so that m can be calculated from the definition of the concentration of diamond grains shown in Equation (10) [23], where $C_{1}$ is a dimensionless constant with a value of 0.03, $C_{a}$ is the concentration of abrasive, $S_{a}$ is the length of a single octahedral prism of a single abrasive grain and $\;A_{0}$ is the area of the end face of the diamond grinding head, with a value of 2πr $\times a_{p}$.

(9) $$F_{c}=mF_{n}$$

(10) $$m=C_{1}\frac{C_{a}^{\frac{2}{3}}A_{0}}{S_{a}^{2}}$$

**Figure 6:**
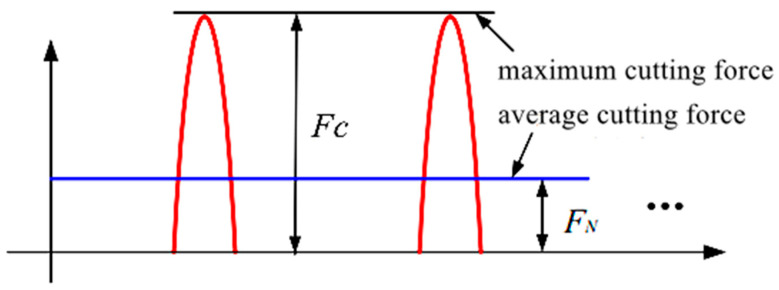
Relationship between maximum cutting force and average cutting force of diamond abrasive grains in longitudinal torsional composite ultrasonic machining.

During the longitudinal torsional compound ultrasonic vibration grinding process, the head and the workpiece are in an intermittent processing state, so that the force exerted by the head is actually pulsed. The pulse waveform during the actual machining can be approximated by a triangular waveform, as shown in Figure 6. According to the momentum theorem, in one vibration cycle, the impulse size of the average cutting force on the grinding head is equal to the impulse value of the maximum impact force, and the relationship between the cutting force $F_{N}$ applied to the grinding head as a whole and the cutting force $F_{c}$ applied to a single abrasive grain on the grinding head is shown in Equation (11).

(11) $$F_{N}\cdot \frac{1}{f}=mF_{c}\cdot \frac{\Delta t}{2}$$

The volume of material removed from the head can be derived from the sum of the volumes of material removed from all abrasive grains at the end of the head. Therefore, the material removal volume for longitudinal torsional composite ultrasonic vibrational grinding of brittle materials in the brittle fracture removal mode can be derived from Equation (7).

(12) $$V_{2}=m\cdot f\cdot V=2K\cdot m\cdot C_{l}\cdot C_{h}\cdot L\cdot f$$

Alternatively, $V_{2}$ can be calculated based on process parameters, including feed rate, cut depth and cut width, as shown in Equation (13), where $v_{c}$ is the feed rate of the cut tool in mm/s, $a_{e}$ is the width of the cut in mm and $a_{p}\;$is the depth of cut in mm.

(13) $$V_{2}=v_{c}\cdot a_{e}\cdot a_{p}$$

Combining the above equations, it can be deduced that the grinding head is subjected to cutting forces during machining as shown in Equation (14), where K is the scale factor, the value of which can be determined experimentally.

(14) $$F=K\cdot {\left[\frac{H^{35}\cdot K_{IC}^{12}\cdot {\left(1-v^{2}\right)}^{6}\cdot C_{a}^{4/3}\cdot v_{c}^{24}\cdot a_{e}^{24}\cdot a_{p}^{24}}{\xi \cdot \cot^{17}\left(\alpha_{0}∕2\right)\cdot E^{21}\cdot S_{a}^{4}\cdot A^{2}\cdot n^{24}\cdot r^{24}}\right]}^{1/26}$$

### 2.4. Determination of the Cut Force Model Coefficient K

This section focuses on determining the specific value of K, by setting different process parameters, to obtain the final cut force model. The validity of the established model is then experimentally verified by testing other different process parameters to verify the cut force values predicted by the model and the actual cut force values during the grinding process.

The experimental material used for this study is an atmospheric-pressure-fired SiC ceramic material, whose physical properties are shown in Table 1. The tool used for the experiment was a diamond grinding head with a base material diameter of 6 mm, a diamond grain size of #200 and a wall thickness of 0.4 mm, where the top angle α of the single grain was 90°, and the average size $S_{a}$ was 130 μm. The experimental material was mounted on the force-measuring instrument through a special fixture, and the amplitude of ultrasonic vibration at the end of the grinding head was measured using a Keyence LK-H020 laser vibrometer before the experiment, where the longitudinal amplitude $A_{1}$ was 3.85 μm, and the torsional amplitude $A_{2}$ was about 7.65 μm. The processing parameters are shown in Table 2.

The value of the coefficient *K* was obtained by the least squares method as 1.025 × 10^4^, and the prediction model of the cutting force during the longitudinal torsional compound ultrasonic vibration grinding of SiC ceramic material was obtained by substituting it into Equation (14).

**Figure 7:**
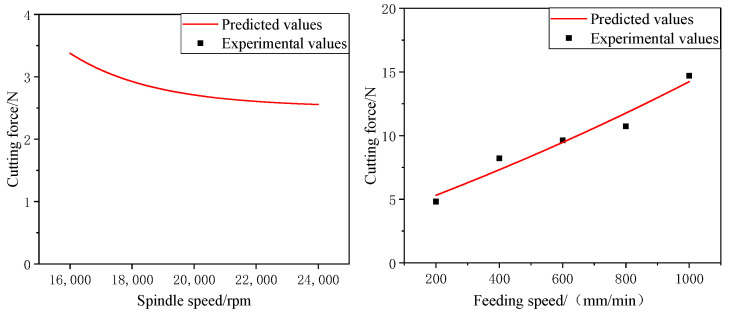
Comparison of predicted and experimental values.

A comparison of the predictions of the above model with the experimental results is shown in Figure 7. From the figure, it can be seen that the predicted values and the experimental results values change in the same trend, and the error value of the model stays within 15%. From the experimental validation results, we can see that the cut force prediction model developed above can effectively predict the cut force during longitudinal torsional compound ultrasonic vibration grinding of SiC ceramic materials. The errors were mainly caused by the uneven distribution of diamond head grains and the size of individual grains during the actual milling process, the use of coolant during the milling process and machining errors of the machine tools.

## 3. Experimental Details

In this work, a series of ultrasonic-vibration-assisted grinding experiments with longitudinal torsion and scratching were performed on SiC ceramics, where scratch surface quality, cutting force, surface roughness, and sub-surface cracks were measured. Based on the experimental results, we analyze the pattern of the impact of the treatment parameters on the various metrics.

### 3.1. Experiment Setup

**Figure 8:**
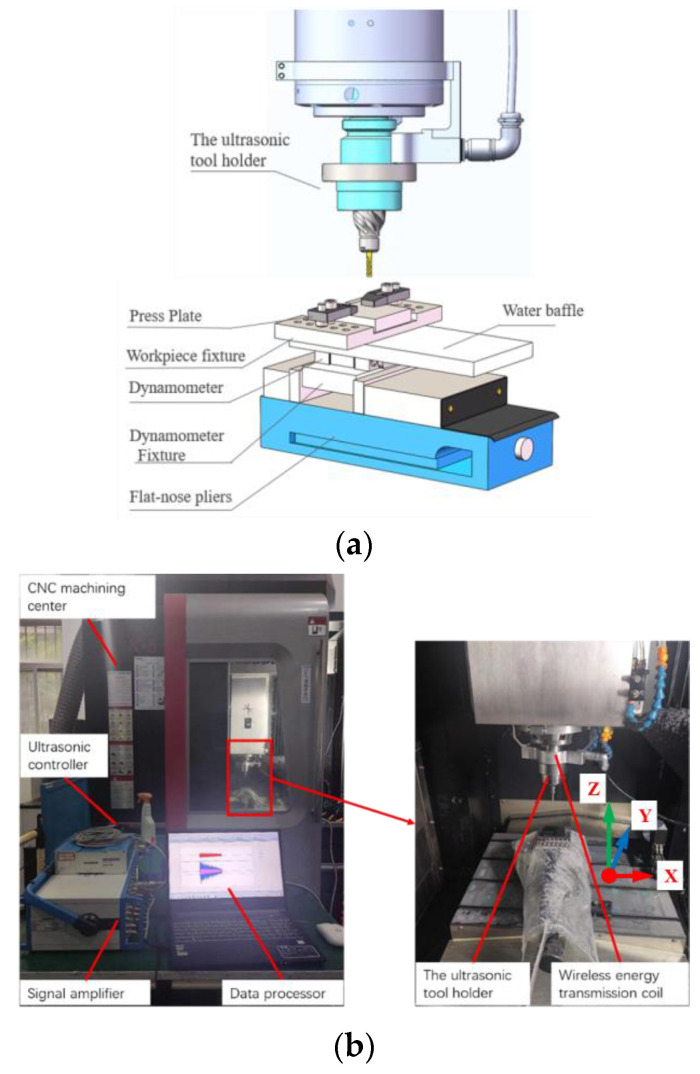
Experimental setup and schematic diagram. (**a**) Schematic mounting method of the experiment setup. (**b**) Experimental setup.

A longitudinal torsional compound ultrasonic-vibration-assisted scratch test and a grinding test were designed. The effect of longitudinal torsional compound ultrasonic vibration on the removal form of SiC ceramic materials was studied by comparing the removal form of a single diamond abrasive grain subjected to longitudinal torsional compound ultrasonic vibration with normal processing. The longitudinal twist compound ultrasonic vibration grinding test was mainly set up with different grinding parameters (spindle speed, feed rate and depth of cut) to compare ultrasonic grinding of SiC and ordinary grinding of SiC, and to observe the cutting force during grinding and the surface quality, surface roughness and sub-surface cracks of the workpiece after grinding. The longitudinal torsional compound ultrasonic vibration grinding SiC test and the scratching SiC test in this paper were completed on the ultrasonic grinding system. As shown in Figure 8, the system consists of a CNC machining center, an ultrasound controller, a wireless energy transfer coil, an ultrasound tool holder, a force measurement instrument, a signal amplifier and a signal analyzer. The radio energy transfer coil is mounted on the outer ring of the spindle of the machine tool and is primarily used to receive the ultrasonic signal from the ultrasonic controller and transmit the signal to the ultrasonic tool holder via magnetic susceptibility.

### 3.2. Experiment Design

#### 3.2.1. Longitudinal Torsion Compound Ultrasonic Vibration Scratch Experiment

**Figure 9:**
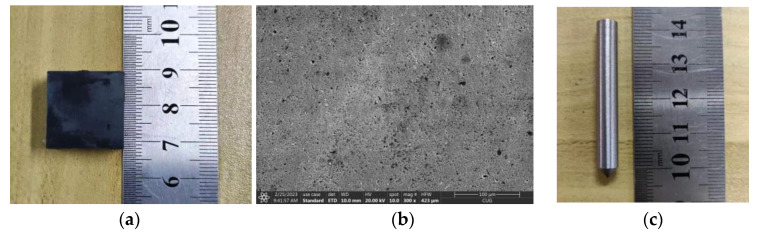
Experimental materials: (**a**) polished SiC ceramic object, (**b**) surface morphology of SiC ceramics after polishing and (**c**) physical drawing of the scrubbing head.

The material of this scratch test is SiC ceramic with dimensions of 20 mm × 20 mm × 10 mm, as shown in Figure 9a. Due to the small size of single-grain diamond abrasive scratch, in order to facilitate the observation of the scratch surface morphology after the test, the surface of the SiC ceramic is polished before the test starts, as shown in Figure 9b. Before the experiment, the force gauge holder was polished to ensure levelness, then the SiC ceramic was glued to the force gauge holder by heat-melt adhesive, and the holder was secured to the force gauge by hexagonal screws. The flatness of the SiC ceramic surface is ensured by a micrometer. As shown in Figure 9c, the test tool is a diamond grinding head with a single abrasive grain, which is fixed to the substrate material by welding. The substrate material for the head is aluminum alloy with a diameter of 6 mm and a length of 40 mm. The taper angle of the end of the diamond grain is 90°, and the radius of the arc is about 100 μm.

The table of parameters for the ab initio test is given in Table 3. Before the experiment, the ultrasonic amplitude of the bottom of the single abrasive diamond tool was measured by the laser displacement sensor to be 8.07 μm for A1 and 5.07 μm for A2. In order to observe the effect of different cutting depths on the removal form the SiC ceramic material, the table was rotated so as to raise the left side of the SiC ceramic by 30 μm, and the single abrasive diamond tool started scratching from the right side of the workpiece, so the scratching depth was 0–0.030 mm. The scratch feed rate was 50 mm/min, the scratch length was 20 mm, the scratch tests were performed at a spacing of 2 mm and the tests were repeated three times for each set of parameters. After the test, the surface shape of the scratch was observed using a scanning electron microscope (SEM).

#### 3.2.2. Longitudinal Torsion Compound Ultrasonic Vibration Grinding Experiment

**Figure 10:**
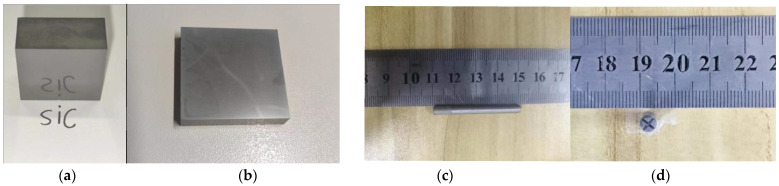
SiC ceramics and physical diagram of grinding head: (**a**) front view of SiC ceramic, (**b**) top view of SiC ceramic, (**c**) front view of tool and (**d**) top view of tool.

The material used in this experiment is atmospheric-pressure-sintered SiC ceramic. As shown in Figure 10a,b, the surface of the SiC material is polished to achieve the mirror effect, and its size is (length × width × height) 50 × 50 × 10 mm. The grinding heads used in this test are shown in Figure 10c,d. The grinding grains are electroplated diamond grains, the diameter of the grinding head is 4 mm, the length is 40 mm (the length of the electroplated diamond grains is 8 mm) and the graininess of the grinding grains is #200. There is a 0.5 mm wide and 1 mm deep cross groove at the bottom of the grinding head, because the linear speed of the center point of the grinding head is 0 during the grinding process, the cutting heat accumulation is too fast and the chips are easy to stick at the bottom of the grinding head, resulting in accelerated wear of the grinding head. So, a cross groove was designed at the bottom of the grinding head to avoid wear of the head due to a linear velocity of 0 at the center point.

The effect laws of different process parameters (spindle speed, feed rate, depth of cut and ultrasonic vibration) on cutting force, surface quality and subsurface damage were investigated by setting up single-factor experiments. This is shown in Table 4.

A three-factor, five-level orthogonal test was designed with spindle speed, feed rate and depth of cut as factors to study the optimal process parameters for the improvement ratio of cutting force for longitudinal torsional compound ultrasonic vibration grinding compared with normal grinding, and the orthogonal test table is shown in Table 5.

## 4. Results and Discussion

### 4.1. Scratch Experimental Results and Analysis

#### 4.1.1. Analysis of the Effect of the Ultrasound Amplitude on The Scratching Force

**Figure 11:**
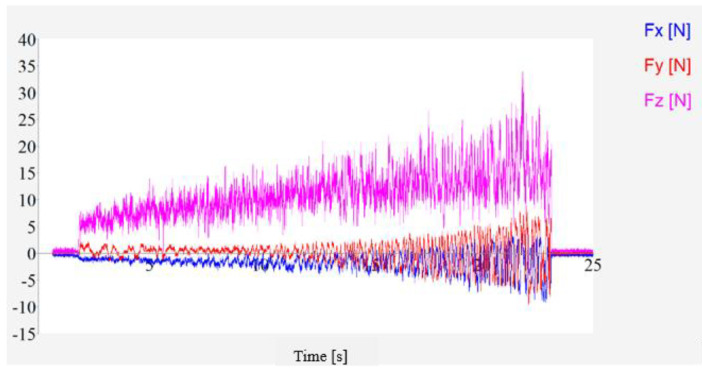
Scratching force with time.

Figure 11 shows the variation in the cutting force over time for the scratch test, during which the grinding head is fed continuously in the negative direction of X in the machine coordinate system. From the figure, it can be seen that the cuts in each direction gradually increase, which is due to the fact that the depth of the cut increases, the amount of material removed from the tip per unit time increases and the tip wear increases, resulting in an increase in the cutting force.

**Figure 12:**
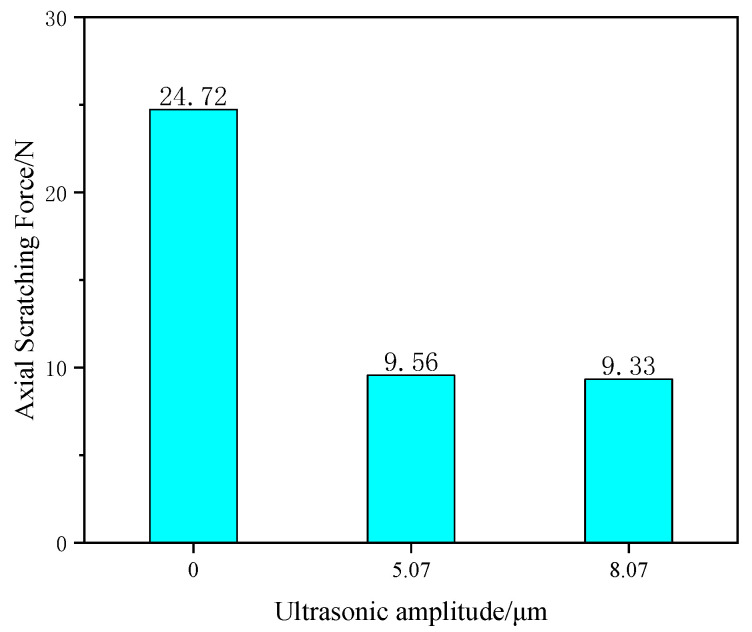
Comparison of scratching force.

Figure 12 shows the graphs of the scratching force (mainly considering the axial force) for ultrasonic scratching SiC ceramics with an ultrasonic amplitude of 5.07 μm and 8.07 μm and for ordinary scratching SiC ceramics. This is because the high-frequency vibration of ultrasonic longitudinal during the scratching process effectively reduces the contact time between the tool tip and the material, realizes the cutting separation in the axial direction, greatly reduces the accumulation of cutting heat during the scratching process, reduces the wear of the tool tip and thus greatly reduces the cutting force. And the ultrasonic longitudinal vibration is conducive to strengthening the “hammering” effect of diamond abrasive grains on the surface of the SiC ceramic material, which makes the removal of material easier and thus reduces the scratching force in the scratching process. The improvement of cutting force by different ultrasonic amplitude is not obvious because, from the principle of ultrasonic vibration, reducing the cutting force can both realize the cutting over the scratching process and realize the “hammering” effect on the material, so the difference of scratching force by different ultrasonic amplitude is not big.

#### 4.1.2. Analysis of the Effect of Ultrasonic Amplitude on the Quality of Scratched Surfaces

**Figure 13:**
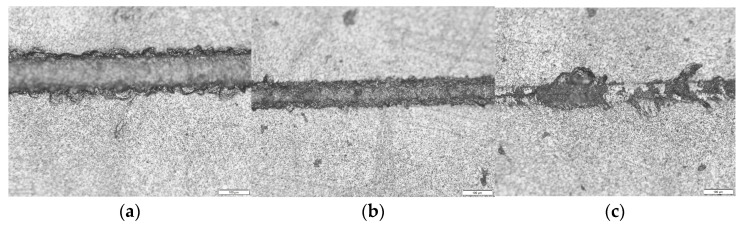
Scratch surface quality diagram: (**a**) ultrasonic amplitude 8.07 μm, (**b**) ultrasonic amplitude 5.07 μm and (**c**) common scratch.

**Figure 14:**
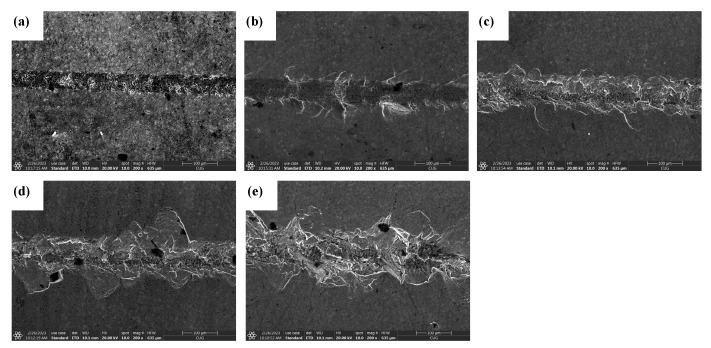
Surface morphology of common scratches at different locations. (**a**) show the surface morphology at the start of the scratch; (**b**–**d**) show the sur-face morphology at the middle of the scratch; (**e**) show the sur-face morphology at the end of the scratch.

**Figure 15:**
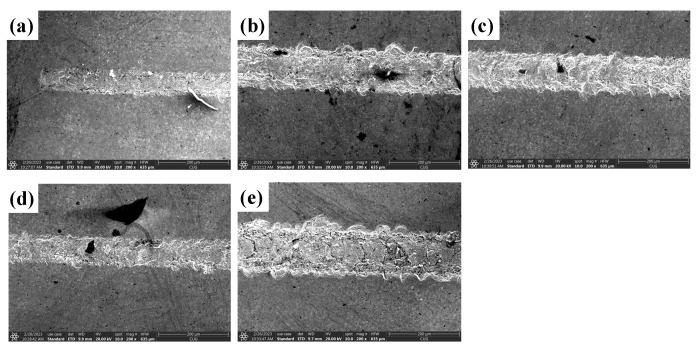
Surface morphology of ultrasound (ultrasound amplitude 5.07 μm) scratches at different positions. (**a**) show the surface morphology at the start of the scratch; (**b**–**d**) show the sur-face morphology at the middle of the scratch; (**e**) show the sur-face morphology at the end of the scratch.

**Figure 16:**
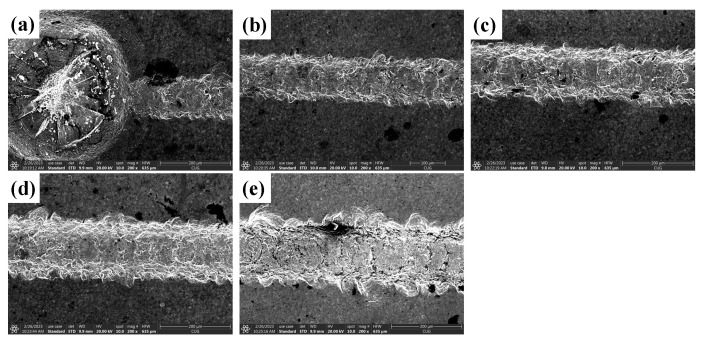
Surface morphology of ultrasound (ultrasound amplitude 8.07 μm) scratches at different positions. (**a**) show the surface morphology at the start of the scratch; (**b**–**d**) show the sur-face morphology at the middle of the scratch; (**e**) show the sur-face morphology at the end of the scratch.

Figure 13 shows the surface morphology of SiC for common scratch and ultrasonic scratch tests taken by optical microscopy, and Figure 14, Figure 15 and Figure 16 show the surface morphology of SiC for common scratch and ultrasonic scratch tests taken by scanning electron microscopy at different positions. Figure 14a, Figure 15a and Figure 16a show the surface morphology at the start of the scratch; Figure 14b–d, Figure 15b–d and Figure 16b–d show the surface morphology at the middle of the scratch; Figure 14e, Figure 15e and Figure 16e show the surface morphology at the end of the scratch. From Figure 14, Figure 15 and Figure 16, it can be seen that the deformation removal process of the surface of ordinary scratching and ultrasonic-vibration-assisted scratching materials changes from plastic removal to brittle removal as the depth of the surface of SiC scratched by a single grain diamond abrasive bit gradually increases. From Figure 14, Figure 15 and Figure 16, it can be seen that the brittle fracture of the surface of SiC by ordinary scratching is most pronounced, with a large number of cracks and brittle spallation at the bottom and sides of the scratch; comparing the surface of ultrasonic scratching with different amplitudes, it can be seen that the brittle removal is also obvious when the amplitude is 8.07 μm, and there are small brittle fracture pits and brittle spalling phenomena at the edges; the brittle spalling phenomenon on the surface of the material is the smallest when the amplitude is 5.07 μm. From the comparison results, it can be seen that (1) ultrasonic scratching of SiC can effectively reduce the brittle flaking phenomenon caused by brittle removal and (2) the larger the ultrasonic amplitude is not better, and the brittle removal of SiC ceramics starts to increase at larger amplitudes.

#### 4.1.3. Analysis of the Effect of Ultrasonic Amplitude on the Depth of Cut of Brittle–Plastic Transformation

**Figure 17:**
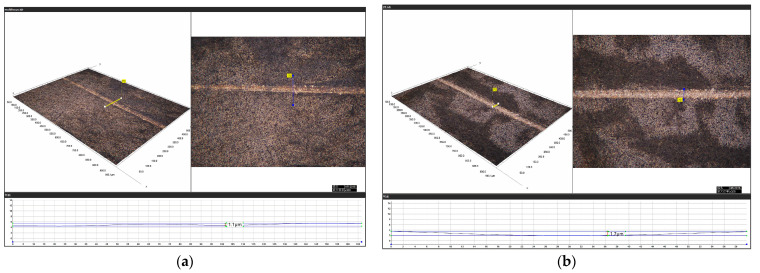

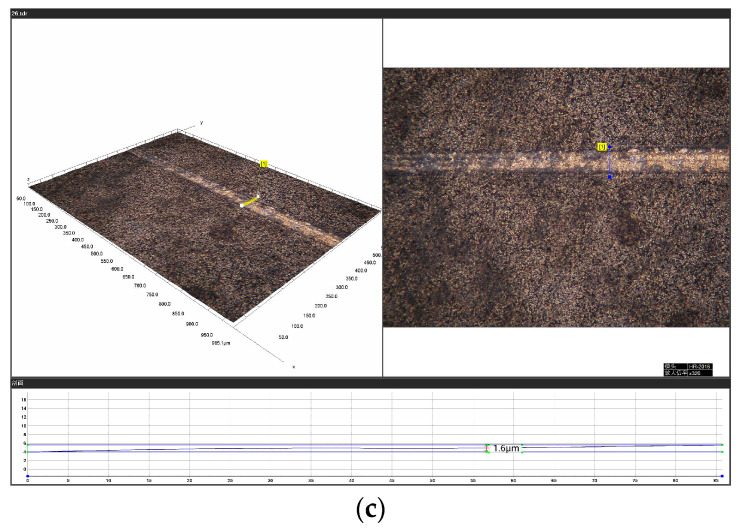
Measurement of the depth of cut of the brittle–plastic transformation: (**a**) CG, (**b**) LTUVG-5.07 μm and (**c**) LTUVG-8.07 μm. The icons in the lower right corner of the figure represent the lens model and magnification.

**Figure 18:**
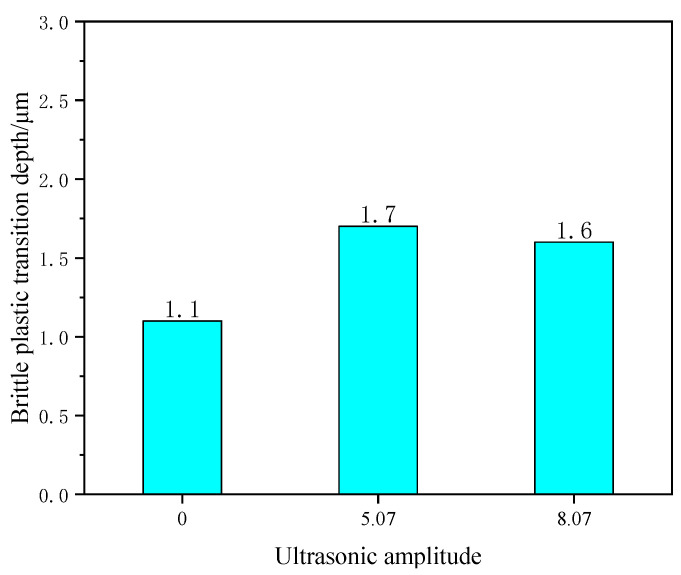
Comparison of depth of cut of brittle–plastic transformation between ordinary scratching and ultrasonic scratching.

The bottom panel of Figure 17 shows depth-cut measurements of ordinary scratch and ultrasonic-vibration-assisted scratch brittle–plastic transitions taken by ultra-deep field microscopy. Among them, Figure 17a shows the height measurement of brittle–plastic transformation by ordinary scratching, and Figure 17b,c show the height measurement of the brittle–plastic transformation by scratching with an ultrasonic amplitude of 5.07 μm and 8.07 μm, respectively. As shown in Figure 18, it can be seen that the brittle–plastic transition depth of cut is about 1.1 μm for ordinary scratching, 1.7 μm for 5.07 μm scratching, 1.6 μm for 8.07 μm scratching and 35.29% for 5.07 μm scratching compared with ordinary scratching. At 8.07 μm, the depth of cut of brittle–plastic transformation on the scratched surface increased by 31.25% compared with that of ordinary scratches.

### 4.2. Cutting Force

**Figure 19:**
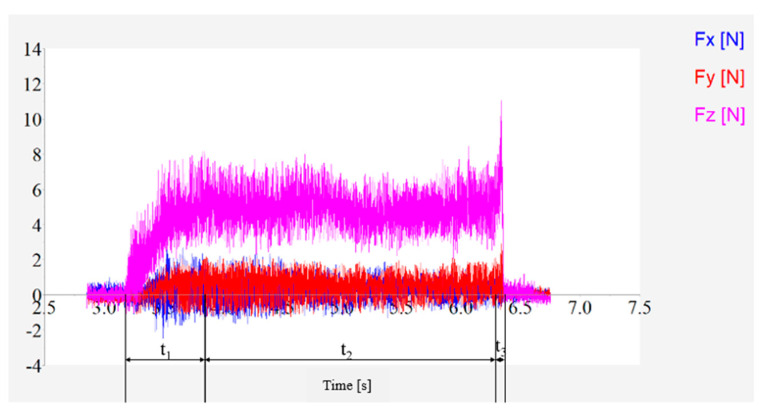
Variation of grinding cut force with time.

As shown in Figure 19, the cutting force in the X, Y and Z directions varies with time during the grinding process, and the grinding head is always fed in the negative direction of X in the machine coordinate system during the grinding process. The figure shows that the shear force in the X, Y and Z directions increases rapidly with time, then stabilizes and finally vanishes. In particular, the cut force is smaller in the X and Y directions and largest in the Z direction, indicating that the main cut force is axial during the grinding process. The raw data of the machining force are divided into three main stages depending on the machining state. One of the cutting stages is the grinding head plunge stage, where the magnitude of the force in the three directions in stage one is continuously increased. This is due to the fact that the bottom of the mill head first touches the workpiece at the beginning of stage 1, when the material removal mainly relies on the bottom edge of the mill head. As the machine is fed in the X direction, the actual cutting radius of the tool increases, resulting in increased forces in all three directions. At the end of stage one, the radius of the grinding head is fully cut into the cutting stage two; at this time, the side cutting area and the bottom cutting area cutting volume remains unchanged, thus the size of the cutting force in all directions tends to stabilize, so in the subsequent analysis of the cutting force, data are selected as the main analysis area of stage two. As the processing continues, the mill head penetrates the workpiece, and the milling process enters the cutting phase three. The third stage belongs to the cut-out stage of the mill. As the workpiece is penetrated, the cutting thickness of the bottom edge is 0, resulting in a rapid decrease in the cutting force in the Z direction, but because there is still uncut material in the side wall, the side edge keeps contact with the workpiece, and the material removal process of the side edge has a parting force in the Z direction, so the cutting force in the Z direction does not return to zero immediately, while the cutting force in the X and Y directions decreases more slowly than that in the Z direction. After the grinding head has completely penetrated the workpiece, the grinding process ends with zero cutting force in each direction.

#### 4.2.1. Effect of Spindle Speed on Cutting Force

**Figure 20:**
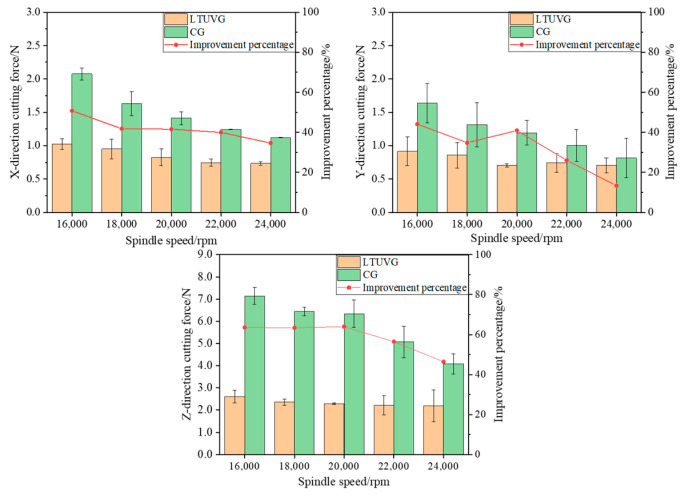
The effect law of spindle speed on cutting force.

From Figure 20, it can be seen that with the increase in spindle speed, the cutting forces in all three directions of normal grinding gradually decreased, the cutting forces in all three directions of longitudinal torsional compound ultrasonic vibration grinding did not change significantly and the cutting forces in all three directions of longitudinal torsional compound ultrasonic vibration grinding decreased in proportion compared with the cutting forces in all three directions of normal grinding. The cutting force in the X direction was reduced by 34.63% to 50.76% by longitudinal compound ultrasonic vibration grinding of SiC. The cutting force in the Y direction was reduced by 13.35 to 44.08%. The cutting force in the Z direction is reduced by 46.42% to 63.57% compared to the case of ordinary ground SiC. This is because an increase in spindle velocity involves more abrasive grains with the same material removal volume, which reduces abrasive head wear and cutting heat, thus effectively reducing the cutting force. In the longitudinal torsional compound ultrasonic grinding process, the increase in the spindle speed will reduce the number of vibrations of the abrasive grains in one rotation cycle, resulting in a reduction in the spatial trajectory of the ultrasonically processed abrasive grains, a reduction in the material removal volume of the abrasive grains and the scratching trajectory of the abrasive grains with previous repetition, so the percentage of improvement on cutting force is reduced.

#### 4.2.2. Effect of Feed Rate on Cutting Force

**Figure 21:**
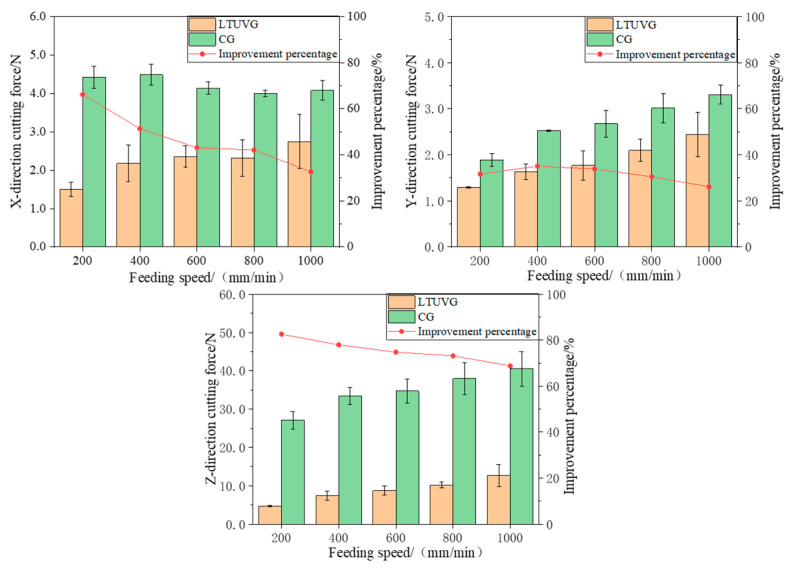
Effect law of feed rate on cutting force.

From Figure 21, it can be seen that with the increase of feed rate, the cutting force in all three directions of normal grinding increases, and the cutting force in all three directions of longitudinal twist compound ultrasonic vibration grinding also increases, and the cutting force in the X and Z directions of longitudinal twist compound ultrasonic vibration grinding decreases compared with the cutting force in the X and Z directions of normal grinding, while the cutting force in the Y direction decreases after increasing first. The cutting forces in the X direction of longitudinal torsion compound ultrasonic vibration grinding of SiC were reduced by 32.63–66.18%, 26.19–35.08% in the Y direction and 68.82–82.59% in the Z direction compared with the cutting forces in the X direction of ordinary grinding of SiC. This is because with the increase of feed speed, the material removal of abrasive grains per unit time increases, and the excessive feed speed also causes the abrasive grains to wear more, which further increases the grinding force. The grinding force of the longitudinal torsion compound ultrasonic grinding process is always smaller than that of the normal process, and the ultrasonic vibration will make the spatial trajectory length of the grinding grain smaller than that of the normal process, thus reducing the grinding force and the periodic contact separation of the grinding head from the workpiece material, which further reduces the grinding force. As the feed rate increases, the number of vibrations of the grinding grain in one cycle decreases, and the spatial trajectory of the grinding grain is closer to that of normal grinding, which leads to a proportional reduction in the cutting force.

#### 4.2.3. Effect of Depth of Cut on Cutting Force

**Figure 22:**
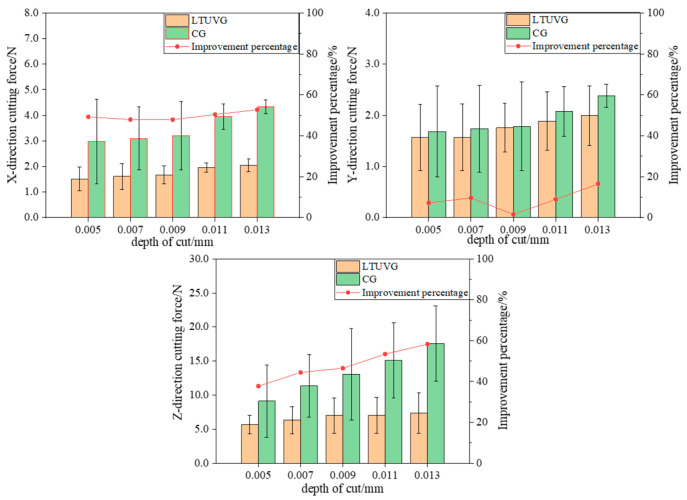
The effect of depth of cut on cutting force.

From Figure 22, it can be seen that as the depth of cut increases, the cutting forces in all three directions of normal grinding and the cutting forces in all three directions of longitudinal compound ultrasonic vibration grinding increase, and the cutting forces in the X and Y directions of longitudinal compound ultrasonic vibration grinding decrease and then increase compared to the cutting forces in the X and Y directions of normal grinding, and the cutting forces in the Z direction of longitudinal compound ultrasonic vibration grinding increase compared to the cutting forces in the Z direction of normal grinding. The reduction of the cutting force in the Z direction of the longitudinal torsion compound ultrasonic vibrational grinding is increased compared to the normal grinding. The cutting forces in the X direction of longitudinal compound ultrasonic vibration grinding of SiC were reduced by 47.83–52.72%, the cutting forces in the Y direction were reduced by 2–16.45% and the cutting forces in the Z direction were reduced by 11.70–61.76% compared with those of ordinary grinding of SiC. This is because when the grinding depth is small, the amount of material removed by the tool is also small, and therefore the grinding force is smaller. When the depth of grinding is increased, the number of abrasive grains involved in grinding increases, the amount of material removed becomes larger, the volume of material removed becomes larger, the grinding force increases continuously, the cutting heat increases and the wear on the head increases. The longitudinal torsion compound ultrasonic vibration grinder is in intermittent contact with the workpiece, which helps to reduce the cutting heat and wear on the grinder head, so that the cutting force does not increase significantly. As the depth of cut increases, the advantage of longitudinal torsion compound vibration grinding is more obvious compared with normal grinding, so the percentage of reduction in the cutting force increases.

### 4.3. Surface Roughness

#### 4.3.1. Effect of Spindle Speed on Surface Roughness

**Figure 23:**
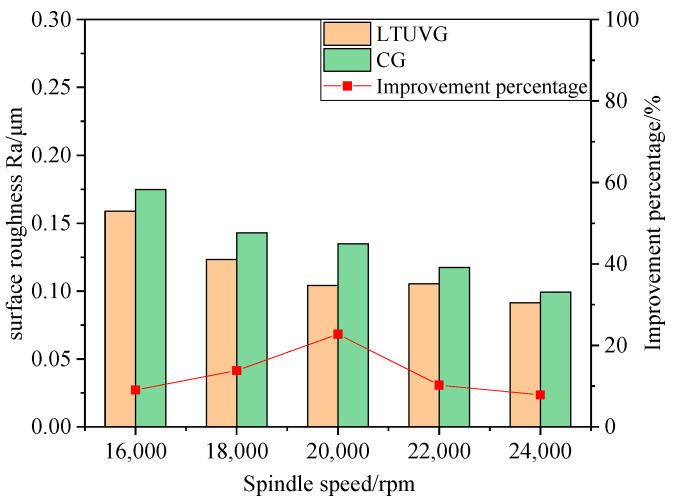
The effect law of spindle speed on surface roughness.

The surface roughness Ra of the SiC ceramics treated with longitudinal torsional ultrasonic vibrations is lower than the surface roughness of common grinding, as shown in Figure 23, and that the surface roughness Ra values of both longitudinal compound ultrasonic vibration grinding and ordinary grinding decrease with increasing spindle velocity. The best improvement in roughness was achieved at a spindle speed of 20,000 rpm. This is because as the principal axis velocity increases, the number of abrasive grains involved in material removal per unit time increases, which is more favorable for material removal, and thus the surface roughness decreases.

#### 4.3.2. Effect of Feed Rate on Surface Roughness

**Figure 24:**
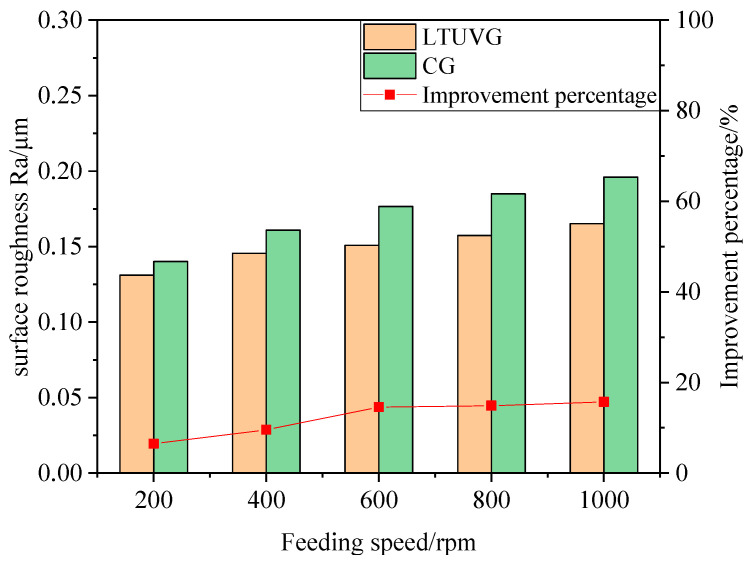
The effect law of feed speed on surface roughness.

As shown in Figure 24, the surface roughness Ra of SiC ceramics treated with longitudinal twist compound ultrasonic vibration grinding is lower than that of ordinary grinding. As the feed rate increases, the surface roughness Ra increases for both plain and longitudinal twist compound ultrasonic vibration grinding, and the percentage of surface roughness improvement for longitudinal twist compound ultrasonic vibration grinding increases from 6.47 percent to 15.73 percent. This is because as the feed rate increases, the amount of material removed per unit time involving abrasive grains increases, and the wear of abrasive grains increases, thus increasing the surface roughness Ra value.

#### 4.3.3. Effect of Depth of Cut on Surface Roughness

**Figure 25:**
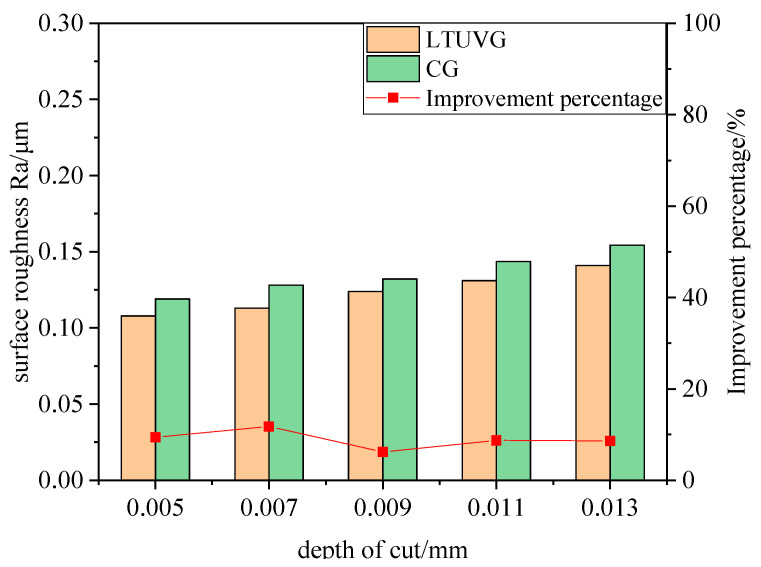
The effect of depth of cut on surface roughness.

As shown in Figure 25, the surface roughness Ra of SiC ceramics processed by longitudinal twist compound ultrasonic vibration grinding is lower than that of ordinary grinding, and the surface roughness Ra values of both longitudinal twist compound ultrasonic vibration grinding and ordinary grinding increase with the increase of cutting depth, and the improvement ratio of longitudinal twist compound ultrasonic vibration grinding on surface roughness does not change much and is stable at about 10%. This is because as the cutting depth increases, the volume of material removed by the abrasive grains per unit time increases, the cutting force increases and the abrasive wear increases, thus causing the surface roughness to increase.

In summary, it can be seen that the surface roughness of SiC Ra by ultrasonic vibration grinding with longitudinal torsion compounds is lower than that by ordinary grinding. This is because ultrasonic processing realizes intermittent processing, which reduces the cutting force and abrasive wear, and the “hammering” effect of ultrasonic vibration is more conducive to material removal, thus reducing the surface roughness.

### 4.4. Subsurface Crack28

**Figure 26:**
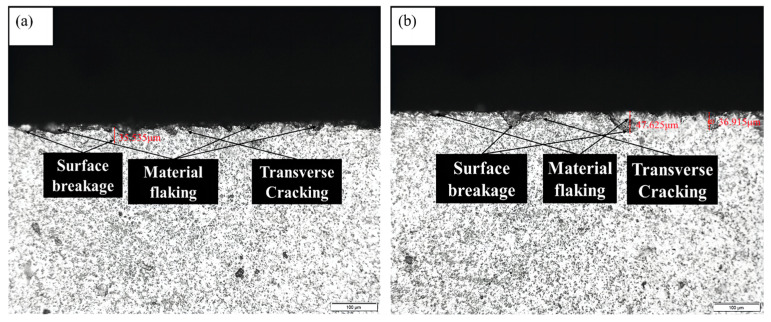
Subsurface morphology: (**a**) LTUVG and (**b**) CG.

Figure 26 shows the subsurface morphology of SiC ceramics processed by longitudinal torsion compound ultrasonic vibration grinding and ordinary grinding with the same process parameters. In order to compare the effect of the two treatments on the quality of the subsurface, the maximum depth of the subsurface was used as an index to compare the longitudinal torsional compound ultrasonic vibration grinding treatment with the ordinary grinding treatment.

#### 4.4.1. Effect of Spindle Speed on Subsurface Cracking

**Figure 27:**
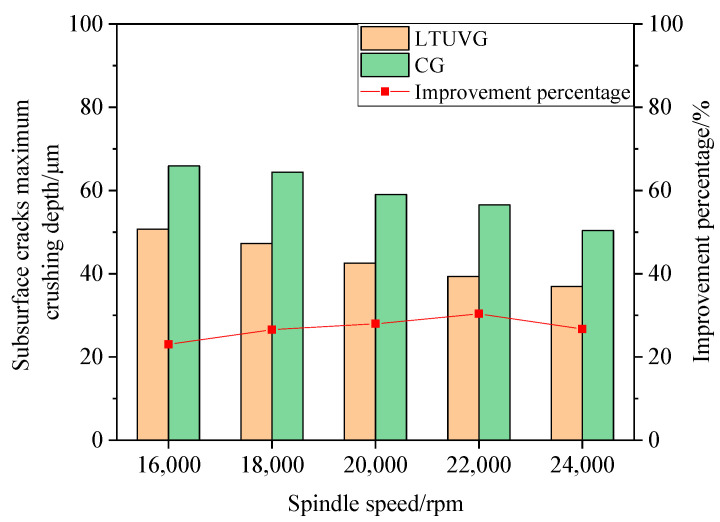
The effect law of spindle speed on subsurface cracks.

Figure 27 shows that the maximum crushing depth of SiC ceramic subsurface wear processed by longitudinal torsion compound ultrasonic vibration grinding is lower than that of ordinary grinding, and the maximum crushing depth of SiC ceramic subsurface wear processed by ordinary grinding and longitudinal torsion compound ultrasonic vibration grinding decreases with the increase in spindle speed. In addition, the improvement ratio of longitudinal torsion compound ultrasonic vibration grinding on the maximum crushing depth increases first and then decreases, from 23.03% to 30.37% and then decreasing to 26.71%, and the improvement ratio was greatest at the spindle speed of 22,000 rpm. This is because the number of abrasive grains involved in the cut per unit time increases with increasing spindle velocity, and the load on a single abrasive grain decreases, which reduces the cutting force and decreases the crack expansion rate.

#### 4.4.2. Effect of Feed Rate on Subsurface Cracking

**Figure 28:**
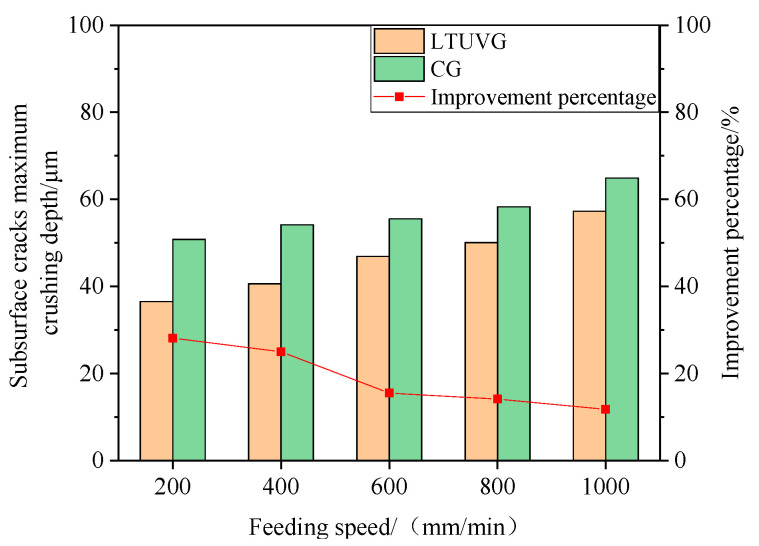
The effect of feed rate on the maximum crushing depth of subsurface cracks.

Figure 28 shows that the maximum crushing depth of subsurface wear of SiC ceramics processed by longitudinal twist compound ultrasonic vibration grinding is lower than that of ordinary grinding, and with the increase of feed speed, the maximum crushing depth of subsurface wear of SiC ceramics processed by ordinary grinding and longitudinal twist compound ultrasonic vibration grinding increases, and the improvement ratio of maximum crushing depth by longitudinal twist compound ultrasonic vibration grinding decreases from 28.13% to 11.78%. This is because as the feed speed increases, the contact area of a single abrasive grain with the material increases per unit time, and the scratching force increases, which leads to the crack opening more easily and thus the maximum crushing depth increases. However, as the feed rate increases, the number of ultrasonic cut separations per unit time decreases, and the effect of the ultrasonic vibrations decreases, hence the improvement ratio decreases.

#### 4.4.3. Effect of Depth of Cut on Subsurface Cracking

**Figure 29:**
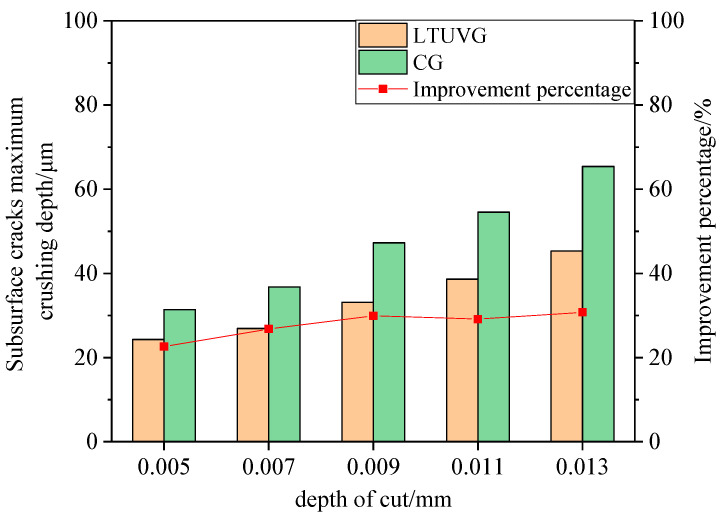
The effect of depth of cut on subsurface cracking pattern.

Figure 29 shows that the maximum crushing depth of subsurface wear of SiC ceramics processed by longitudinal torsion compound ultrasonic vibration grinding is lower than that of ordinary grinding, and the maximum crushing depth of subsurface wear of SiC ceramics processed by ordinary grinding and longitudinal torsion compound ultrasonic vibration grinding increases with the increase of cutting depth, and the percentage of improvement of maximum crushing depth by longitudinal torsion compound ultrasonic vibration grinding increases from 22.63% to 30.75%. This is because as the cutting depth increases, the increase in the amount of material removed per unit time of the abrasive grain leads to an increase in the grinding force during the machining process, which accelerates the crack expansion rate. In contrast, in ultrasonic grinding, the increase in cutting force is less pronounced due to the separation effect of the ultrasonic cuts, which has little effect on the crack expansion rate and thus increases the improvement ratio.

### 4.5. Results and Analysis of Multi-Factor Experiments

#### 4.5.1. Orthogonal Experimental Protocol

For the purpose of investigating the main factors affecting the percentage improvement of the cutting force with longitudinal torsional compound ultrasound grinding compared to normal grinding, a three-factor, five-level orthogonal test table was designed as shown in Table 6.

#### 4.5.2. Orthogonal Experiment Results

The analysis of extreme variance is one of the most common methods for analyzing results in orthogonal tests. This analysis approach considers that the differences in the levels of the factors are due to the factors themselves. Table 7, Table 8 and Table 9 show the polar difference analysis tables for the percentage improvement in cutting force for longitudinal torsional compound ultrasonic vibration grinding compared to normal grinding in the X, Y and Z directions, respectively. The larger the difference between the extrema of each factor, that is, the larger the value of R, the stronger the ability of the factor to influence the cutting force. According to the R-values in Table 7, Table 8 and Table 9, the main factor affecting the improvement ratio of cutting force under the combined effect of three factors is depth of cut, which is in the order of depth of cut > feed rate > spindle speed. This is because the fundamental reason for the improvement of cutting force by ultrasonic vibration grinding is that ultrasonic machining realizes the cutting separation phenomenon and the “hammering” effect of ultrasonic vibration, which facilitates the material removal. The depth of cut directly affects the ability of ultrasonic longitudinal vibration to achieve cutting separation and the “hammering” effect, so it is the main influencing factor. The change of feed speed will change the number of cutting separations per unit time and the number of ultrasonic “hammering” actions, while the spindle speed mainly affects the ultrasonic vibration of the torsional cutting separation phenomenon on the axial cutting separation, and the ultrasonic “hammering” effect is not significant. Therefore, the factors that affect the ratio of cutting force improvement are depth of cut > feed rate > spindle speed, in order.

**Figure 30:**
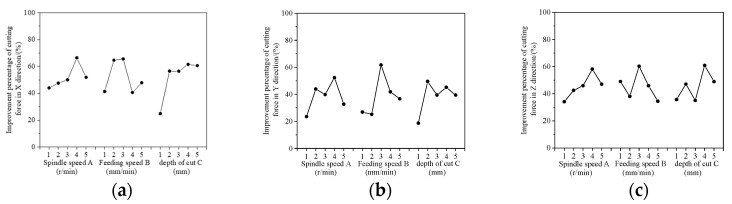
K-value graph of the improvement ratio of cutting force in the X, Y and Z directions. (**a**) X direction. (**b**) Y direction. (**c**) Z direction.

The plots of the K-values for the orthogonal test cut force improvement ratio in the three directions are shown in Figure 30. By analyzing the cutting force improvement index with the K-value graph of each factor and taking the maximum K-value point of each factor, it is known that the level combination of three factors A4B3C4 is the optimal level combination for this test with the cutting force improvement ratio as the index. In other words, the optimal process parameters for the transverse torsion compound ultrasonic vibration grinding of SiC with respect to the cut force reduction ratio are a spindle speed of 22,000 rpm, a feed rate of 600 mm/min and a cut depth of 0.011 mm.

## 5. Conclusions

The properties of the longitudinal torsional ultrasonic vibrations grinding process of SiC ceramics were studied, and the cutting force prediction model was developed. The main results of the study were as follows:

(1) The cutting force of longitudinal torsional ultrasonic scratching was reduced by a maximum of 62.26% compared to that of common scribing, and the depth of brittle–plastic transformation of SiC ceramics was increased by 35.29%.
(2) A cutting force model of the longitudinal torsional ultrasonic vibrations for grinding SiC ceramics was developed. The predicted results were in good agreement with the experimental results, and the maximum error was less than 15%.
(3) The maximum percentage improvement of cutting force was 82.59%, the maximum percentage improvement of surface roughness was 22.78% and the maximum percentage improvement of maximum crushing depth of subsurface cracks was 30.75% after the application of longitudinal torsional ultrasonic vibration.
(4) The cutting force was improved sequentially with cutting depth, feed rate and spindle speed. The optimum process parameters for cutting force improvement were a spindle speed of 22,000 rpm, a feed rate of 600 mm/min and a cutting depth of 0.011 mm.

## Figures and Tables

**Table 1:** Physical property parameters of SiC ceramics.

Elastic Modulus [GPa]	Poisson’s Ratio	Fracture Toughness [MPa·m^1/2^]	Hardness [GPa]
410	0.14	3.9	33

**Table 2:** *K* value to confirm the experimental process parameters.

Spindle Speed [rpm]	Feeding Speed [mm/min]	ap [mm]	ae [mm]	Ultrasonic Amplitude[μm]
16,000/18,000/20,000/ 22,000/24,000	400	0.005	6	*A*1 = 3.85, *A*2 = 7.65
24,000	200/400/600/ 800/1000	0.005	6	0
16,000/18,000/20,000/ 22,000/24,000	400	0.005	6	*A*1 = 8.07, *A*2 = 5.07
24,000	200/400/600/ 800/1000	0.005	6	*A*1 = 8.07, *A*2 = 5.07

**Table 3:** Scratching parameters.

Scribing Speed [mm/min]	Scribing Depth [mm]	Ultrasonic Amplitude [μm]
50	0–0.030	5.07
50	0–0.030	8.07
50	0–0.030	0

**Table 4:** Table of single-factor experiment parameters.

Spindle Speed [rpm]	Feeding Speed [mm/min]	Ap [mm]	Ae [mm]	Ultrasonic Amplitude
16,000/18,000/20,000/ 22,000/24,000	400	0.005	4	$A_{1}=8.07,A_{2}=5.07$/0
24,000	200/400/600/800/1000	0.005	4	$A_{1}=8.07,A_{2}=5.07$/0
24,000	400	0.005/0.007/0.009/ 0.011/0.013	4	$A_{1}=8.07,A_{2}=5.07$/0

**Table 5:** Factor levels of orthogonal experiments.

	Factors	Spindle Speed A [rpm]	Feeding Speed B [mm/min]	ap C [mm]
Level				
1		16,000	200	0.005
2		18,000	400	0.007
3		20,000	600	0.009
4		22,000	800	0.011
5		24,000	1000	0.013

**Table 6:** Orthogonal experiment table.

Experiment Number	Spindle Speed A [rpm]	Feeding Speed B [mm/min]	ap C [mm]
1	16,000	200	0.005
2	16,000	400	0.009
3	16,000	600	0.013
4	16,000	800	0.007
5	16,000	1000	0.011
6	18,000	200	0.013
7	18,000	400	0.007
8	18,000	600	0.011
9	18,000	800	0.005
10	18,000	1000	0.009
11	20,000	200	0.011
12	20,000	400	0.005
13	20,000	600	0.009
14	20,000	800	0.013
15	20,000	1000	0.007
16	22,000	200	0.009
17	22,000	400	0.013
18	22,000	600	0.007
19	22,000	800	0.011
20	22,000	1000	0.005
21	24,000	200	0.007
22	24,000	400	0.011
23	24,000	600	0.005
24	24,000	800	0.009
25	24,000	1000	0.013

**Table 7:** Percentage improvement of cutting force in the X direction.

	Factors	Spindle Speed A [rpm]	Feeding Speed B [mm/min]	ap C [mm]
Levels				
Average value 1		44.07%	41.39%	24.86%
Average value 2		47.64%	64.65%	56.57%
Average value 3		50.05%	65.63%	56.48%
Average value 4		66.45%	40.57%	61.55%
Average value 5		51.92%	47.89%	60.67%
Maximum value		66.45%	65.63%	61.55%
Minimum value		44.07%	40.57%	24.86%
Range		22.38%	25.06%	36.68%

**Table 8:** Percentage improvement of cutting force in the Y direction.

	Factors	Spindle Speed A [rpm]	Feeding Speed B [mm/min]	ap C [mm]
Levels				
Average value 1		23.55%	26.88%	18.67%
Average value 2		43.92%	25.23%	49.54%
Average value 3		39.84%	61.71%	39.47%
Average value 4		52.29%	41.84%	45.20%
Average value 5		32.77%	36.69%	39.48%
Maximum value		52.29%	61.71%	49.54%
Minimum value		23.55%	25.23%	18.67%
Range		28.73%	36.48%	30.87%

**Table 9:** Percentage improvement of cutting force in the Z direction.

	Factor	Spindle Speed A [rpm]	Feeding Speed B [mm/min]	ap C [mm]
Levels				
Average value 1		34.14%	49.04%	35.77%
Average value 2		42.54%	38.05%	47.14%
Average value 3		45.97%	60.30%	35.13%
Average value 4		58.16%	45.92%	60.91%
Average value 5		47.08%	34.58%	48.95%
Maximum value		58.16%	60.30%	60.91%
Minimum value		34.14%	34.58%	35.13%
Range		24.02%	25.72%	25.78%

## Data Availability

Not applicable.

## References

[1] Dai C., Yin Z., Wang P., Miao Q., Chen J. (2021). Analysis on Ground Surface in Ultrasonic Face Grinding of Silicon Carbide (SiC) Ceramic with Minor Vibration Amplitude. Ceram. Int..

[2] Zhang D.Y., Xin W.L., Jiang X.G. (2016). Research Trends of Ultrasonic Machining Technology. J. Electr. Process. Mold.

[3] Ahn B.W., Lee S.H. (2009). Characterization and acoustic emission monitoring of AFM nanomachining. J. Micromech. Microeng..

[4] Goel S., Luo X., Comley P. (2013). Brittle–ductile transition during diamond turning of single crystal silicon carbide. Int. J. Mach. Tools Manuf..

[5] Zhang K., Yin Z., Dai C., Miao Q., Zhang P., Cao Z. (2023). Material Removal Mechanism of SiC Ceramics by Elliptic Ultrasonic Vibration-Assisted Grinding (EUVAG) Using Single Grain. Ceram. Int..

[6] Li L., Wan L., Zhou Q. (2020). Crack propagation during Vickers indentation of zirconia ceramics. Ceram. Int..

[7] Wang D., Liang Q., Xu D. (2023). Research on Damage Characteristics of Ultrasonic Vibration-Assisted Grinding of a C/SIC Composite Material. Sensors.

[8] Wang D., Fan H., Xu D., Zhang Y. (2022). Research on Grinding Force of Ultrasonic Vibration-Assisted Grinding of C/SiC Composite Materials. Appl. Sci..

[9] Dai J., Su H., Hu H., Yu T., Zhou W., Ding W., Ji S., Zheng Y. (2017). The Influence of Grain Geometry and Wear Conditions on the Material Removal Mechanism in Silicon Carbide Grinding with Single Grain. Ceram. Int..

[10] Cao J., Zhang Q. (2019). Material Removal Behavior in Ultrasonic Assisted Grinding of SiC Ceramics. Jixie Gongcheng Xuebao/J. Mech. Eng..

[11] Ding K., Li Q., Zhang C. (2021). Experimental Studies on Material Removal Mechanisms in Ultrasonic Assisted Grinding of SiC Ceramics with a Defined Grain Distribution Brazed Grinding Wheel. Int. J. Adv. Manuf. Technol..

[12] Zhou M., Zhao P. (2016). Prediction of Critical Cutting Depth for Ductile-Brittle Transition in Ultrasonic Vibration Assisted Grinding of Optical Glasses. Int. J. Adv. Manuf. Technol..

[13] Zhang Z., Tong J., Zhao J., Jiao F., Zai P., Liu Z. (2021). Experimental Study on Surface Residual Stress of Titanium Alloy Curved Thin-Walled Parts by Ultrasonic Longitudinal-Torsional Composite Milling. Int. J. Adv. Manuf. Technol..

[14] Xu L. (2019). Study on Surface Quality of Ultrasonic Vibration Grinding of Sic Ceramic.

[15] Agarwal S., Rao P.V. (2008). Experimental Investigation of Surface/Subsurface Damage Formation and Material Removal Mechanisms in SiC Grinding. Int. J. Mach. Tools Manuf..

[16] Cao J., Wu Y., Lu D., Fujimoto M., Nomura M. (2014). Fundamental Machining Characteristics of Ultrasonic Assisted Internal Grinding of SiC Ceramics. Mater. Manuf. Process..

[17] Zeng W.M., Li Z.C., Pei Z.J., Treadwell C. (2005). Experimental Observation of Tool Wear in Rotary Ultrasonic Machining of Advanced Ceramics. Int. J. Mach. Tools Manuf..

[18] Zeng W., Xu X., Pei Z. (2009). Experimental Investigation of Tool Wear in Rotary Ultrasonic Machining of Alumina. Key Eng. Mater..

[19] Ding K., Fu Y., Su H., Gong X., Wu K. (2014). Wear of Diamond Grinding Wheel in Ultrasonic Vibration-Assisted Grinding of Silicon Carbide. Int. J. Adv. Manuf. Technol..

[20] Dong G., Lang C., Li C. (2020). Formation mechanism and modelling of exit edge-chipping during ultrasonic vibration grinding of deep-small holes of microcrystalline-mica ceramics. Ceram. Int..

[21] Sun G., Shi F., Ma Z. (2020). Effects of axial ultrasonic vibration on grinding quality in peripheral grinding and end grinding of ULE. Int. J. Adv. Manuf. Technol..

[22] Marshall D.B., Lawn B.R., Evans A.G. (1982). Elastic/Plastic Indentation Damage in Ceramics: The Lateral Crack System. J. Am. Ceram. Soc..

[23] Bi Z., Tokura H., Yoshikawa M. (1988). Study on surface cracking of alumina scratched by single-point diamonds. J. Mater. Sci..

